# Complete Plastid Genomes of Nine Species of Ranunculeae (Ranunculaceae) and Their Phylogenetic Inferences

**DOI:** 10.3390/genes14122140

**Published:** 2023-11-27

**Authors:** Jiaxin Ji, Yike Luo, Linying Pei, Mingyang Li, Jiamin Xiao, Wenhe Li, Huanyu Wu, Yuexin Luo, Jian He, Jin Cheng, Lei Xie

**Affiliations:** 1 State Key Laboratory of Efficient Production of Forest Resources, School of Ecology and Nature Conservation, Beijing Forestry University, Beijing 100083, China; jx9024@bjfu.edu.cn (J.J.); luoyk@bjfu.edu.cn (Y.L.); xiaojiamin0916@bjfu.edu.cn (J.X.); liwenhe@bjfu.edu.cn (W.L.); wuhuanyu22@bjfu.edu.cn (H.W.); yxluo01@bjfu.edu.cn (Y.L.); hejian@bjfu.edu.cn (J.H.); 2 College of Agriculture and Forestry, Longdong University, Qingyang 745000, China; peilinying0724@163.com; 3 State Key Laboratory of Efficient Production of Forest Resources, College of Biological Sciences and Technology, Beijing Forestry University, Beijing 100083, China; lmylmy2220103@bjfu.edu.cn (M.L.); chengjin@bjfu.edu.cn (J.C.); 4 National Engineering Research Center of Tree Breeding and Ecological Restoration, Beijing Key Laboratory of Ornamental Plants Germplasm Innovation and Molecular Breeding, College of Biological Sciences and Technology, Beijing Forestry University, Beijing 100083, China

**Keywords:** complete plastid genome, next-generation sequencing, phylogeny, positive selection, Ranunculeae, IR contraction

## Abstract

The tribe Ranunculeae, Ranunculaceae, comprising 19 genera widely distributed all over the world. Although a large number of Sanger sequencing-based molecular phylogenetic studies have been published, very few studies have been performed on using genomic data to infer phylogenetic relationships within Ranunculeae. In this study, the complete plastid genomes of nine species (eleven samples) from *Ceratocephala*, *Halerpestes*, and *Ranunculus* were de novo assembled using a next-generation sequencing method. Previously published plastomes of *Oxygraphis* and other related genera of the family were downloaded from GenBank for comparative analysis. The complete plastome of each Ranunculeae species has 112 genes in total, including 78 protein-coding genes, 30 transfer RNA genes, and four ribosomal RNA genes. The plastome structure of Ranunculeae samples is conserved in gene order and arrangement. There are no inverted repeat (IR) region expansions and only one IR contraction was found in the tested samples. This study also compared plastome sequences across all the samples in gene collinearity, codon usage, RNA editing sites, nucleotide variability, simple sequence repeats, and positive selection sites. Phylogeny of the available Ranunculeae species was inferred by the plastome data using maximum-likelihood and Bayesian inference methods, and data partitioning strategies were tested. The phylogenetic relationships were better resolved compared to previous studies based on Sanger sequencing methods, showing the potential value of the plastome data in inferring the phylogeny of the tribe.

## 1. Introduction

The complete plastid genome (plastome) has become an increasingly popular tool for phylogenetic studies in recent years [1,2,3,4]. Plastid is a common organelle found in plant cells that contains its own genome which is typically circular and relatively conserved across plant species [5]. The plastomes are often uniparentally inherited [6] and typically include about 80 protein-coding genes and more than 30 RNA genes [7,8]. The high degree of evolutionary conservation, large amount of data, uniparental inheritance, ability to identify polymorphisms, and easy availability make the plastome an ideal marker for studying phylogenetic relationships among plant taxa at different taxonomic levels [9].

The tribe Ranunculeae comprises 16 to 19 genera and about 650 species distributed worldwide, making it the most representative and diverse group within the buttercup family (Ranunculaceae) [10,11,12,13]. Among all the genera in this tribe, *Ranunculus* stands out as the species-rich genus of the family, with about 650 wild species in the world, whereas all the other genera are small or even monotypic [12]. There are four genera: *Ranunculus* L. (with the inclusion of *Batrachium* (DC.) Gray), *Oxygraphis* Bunge, *Helerpestes* E. L. Greene, and *Ceratocephala* Moench distributed in China, and *Ranunculus* is also the largest one of the tribes with more than 120 wild species in China [10,14]. Plants of Ranunculeae include numerous ornamental and medicinal species, with a particularly rich species diversity in temperate and alpine regions [10,15].

In recent years, numerous molecular phylogenetic studies on the tribe Ranunculeae have been published [11,12,13]. However, all of these studies used a small number of DNA fragments for phylogenetic inference, and their results had inevitable limitations such as low resolution and statistical support due to insufficient phylogenetic signal. The complete plastid genomes of the family Ranunculaceae gained more and more attention in the last few years [16,17,18,19]. Both sequence and structural variations (such as IR expansion/contraction, gene inversion, and gene transposition) in the plastomes of Ranunculaceae showed the potential to yield phylogenetic significance when comprehensive data are available [17]. There is an urgent need to incorporate genomic data to deepen our insights into the phylogeny of Ranunculeae. However, a very small number of the plastid genomes of this tribe have been published up to now.

In this study, the complete plastomes of nine species (eleven samples), representing three genera of Ranunculeae, were assembled using the next-generation sequencing method and reference-guided assembly. We described the bioinformatic characteristics of the plastomes, such as gene content, codon usage, RNA editing sites, repeat sequences, and positive selection. We also compared the synteny of the plastid genome sequences across the family to investigate their plastid genome structural variation and gene order. Finally, combining all the currently available plastome sequences of Ranunculeae species in GenBank, we reconstructed the phylogenetic framework to assess the potential value of the plastome sequences across the tribe. The aims of this study are to: understand the variation of the plastomes across *Ranunculus* and its close allies, to compare the plastome structures (gene order and arrangement) of Ranunculeae with those of the other genera of Ranunculaceae, and to advance the phylogenetic and evolutionary understanding of Ranunculeae.

## 2. Materials and Methods

### 2.1. Plant Sampling and Next-Generation Sequencing

Leaf samples of eleven new accessions representing three genera (*Ceratocephala*, *Halerpestes*, and *Ranunculus*) and nine species of tribe Ranunculeae were collected from field (Table 1). The identification of the specimens was conducted by LX (Lei Xie) and all the vouchers were deposited in the herbarium of Beijing Forestry University (BJFC). In addition, we retrieved all the available complete plastome sequences of tribe Ranunculeae as well as plastomes of its allies in Ranunculaceae from GenBank for comparative and phylogenetic analyses. In total, 11 genera and 33 species (36 samples) of Ranunculaceae (Table 1) were included for different analyses (see below in detail).

For each new sample, about 50 mg of dried leaf tissue was ground for DNA extraction. We used DNA extraction kits (Tiangen Biotech Co., Ltd., Beijing, China) to obtain total genomic DNA. Extracted DNAs were checked by 1.0% agarose gel electrophoresis and then were sent to BerryGenomics (Beijing, China) for library construction and next-generation sequencing (NGS). NGS was run on the Illumina NovaSeq 6000 platform (Illumina Inc., San Diego, CA, USA) to generate paired-end reads of 2 × 150 bp.

### 2.2. Plastid Genome Assembling and Annotating

After obtaining raw reads, we used the FASTX Toolkit (http://hannonlab.cshl.edu/fastx_toolkit, accessed on 18 June 2022) to remove the adaptors and low-quality reads. The plastid genome sequences were then de novo assembled according to our previous study [17]. GetOrganelle (https://github.com/Kinggerm/GetOrganelle, accessed on 15 July 2022) was used with SPAdes 3.10.1 as the assembler [20]. Contigs were connected into larger ones using RepeatFinder option in Geneious v. Prime [21], and when necessary, the gaps were bridged using 100 replicates of Fine Tuning in Geneious Prime [21] to generate complete plastome sequences. The gaps and junctions between IRs and LSC/SSC regions were further verified by Sanger sequencing PCR amplifications. The assembled plastome sequences were then annotated using the Plastid Genome Annotator [22]. Plastid genome circles were drawn using the Organellar Genome DRAW v. 1.3.1 [23].

### 2.3. Comparative Analyses of the Plastomes

The newly sequenced plastomes were aligned and compared with those of the previously published Ranunculaceae species. Geneious Prime [21] and CodonW v. 1.4.2 [24] were used to calculate amino acid frequency and codon usage for the new samples. We checked putative RNA editing sites in protein-coding genes by the PREP-cp suite [25] for the new samples. The plastome sequences across Ranunculaceae were aligned using mVISTA [26] for the synteny analysis. We used LAGAN and Shuffle-LAGAN modes with default parameters to detect possible plastome structural variation. IR expansion/contraction of all the available Ranunculeae samples were checked using IRscope [27]. The nucleotide variability (Pi) of the plastomes of both Ranunculeae and *Ranunculus* (which have the most species) were calculated using a sliding window analysis implemented in DnaSP v. 5 [28].

We searched plastid microsatellites by using software MIcroSAtellite (MISA) [29] with a minimum threshold of ten nucleotides for mononucleotide repeats, five for di-, four for tri-, and three for tetra-, penta-, and hexanucleotide repeats according to our previous study [17]. We also searched forward (F), reverse (R), complement (C), and palindromic (P) oligonucleotide repeats using the REPuter program [30] with a minimum repeat size of 30 bp and similarity value of 90%.

### 2.4. Positive Selection Analysis

All the available Ranunculeae samples (25 species, 28 samples) and outgroups (four species from Trib. Anemoneae) were used for CDS extraction using Geneious Prime [21]. The program CODEML implemented in PAML v. 4.10.6 package [31,32] was applied for the positive selection site analysis. We estimated a single dN:dS ratio (ω) of the entire alignment for the null model. Then, the branch model (model = 2; NSsites = 0) was used to estimate a single ω of all the lineages of tribe Ranunculeae as the foreground, and a different ω of the lineages from the outgroups tribe Anemoneae as the background. Finally, a chi-square distribution was applied to assess the significance of the results. On the other hand, the Bayes Empirical Bayes (BEB) method was also applied to identify specific amino acid sites in genes to calculate posterior probability values (PP). High PP values (P > 0.9) of the codon sites were considered to be positive selection sites [33,34]. According to previous studies, we take the genes with a *p*-value < 0.05 and at least one positively selected site with high PP values as a positive selection gene [35].

### 2.5. Phylogenetic Analysis

The phylogenetic framework was reconstructed for all the available species (28 samples representing 25 species) of tribe Ranunculeae. Previous studies showed that tribe Anemoneae is sister to Ranunculeae in the family [16,17,19], so we chose four samples from Anemoneae as the outgroups. For phylogenetic tree reconstruction, the IRa region was excluded from the analysis. Inversion and translocation regions in tribe Anemoneae were manually adjusted. To investigate potential differences in phylogenetic reconstruction using different partitions, we divided the complete plastome sequences under the following partition strategies. The complete dataset was first separated into coding regions (CDS), intergenic spacer regions (IGS), and introns. Each dataset was further separated by their positions: LSC, SSC, and IR, respectively. We ultimately obtained 13 datasets for phylogenetic analysis. They are the complete plastome, the complete CDS sequence, the complete IGS, the complete intron, the LSC-CDS, the LSC-IGS, the LSC intron, the SSC-CDS, the SSC-IGS, the SSC-intron, the IR-CDS, the IR-IGS, and the IR-intron datasets. Multiple alignments for all the datasets were conducted by MAFFT v. 6.833 [36]. We removed ambiguous alignments using a Python script written in our previous study [17].

For each dataset, the maximum likelihood (ML) and the Bayesian inference (BI) methods were applied for phylogenetic reconstruction. Substitution models and data partitions of the complete plastome dataset were tested by PartitionFinder v2.1.1 [37]. We tested six partitioning schemes for the complete plastome dataset according to previous studies [38]. They are (1) no partitions, (2) by coding and non-coding regions, (3) by positions of LSC, SSC, and IRs, (4) by genes for the CDS and non-coding region as a separate partition, (5) by genes and codon positions for the CDS and non-coding region as separate partition, (6) by the third codon position for the coding region. The Bayesian information criterion (BIC) was applied to assess the best partitioning scheme. The ML analysis was carried out using RAxML v.8.1.17 [39] with the GTR + G model recommended in the user’s manual. We run 500 replicates of resampling analysis to obtain the ML bootstrap support values. The BI analysis was conducted using MrBayes v3.2.3 [40], with the default priors for tree search. Two Markov chain Monte Carlo (MCMC) chains, each with three heated and one cold chain, were independently run for 2,000,000 generations with tree sampling every 100 generations. The first 25% of the trees were discarded as burn-in, and the remaining 75% of trees were then summarized to yield the Bayesian consensus phylogram.

## 3. Results

### 3.1. Plastome Characterization of Ranunculeae Genera and Species

We obtained up to 12 Gb raw NGS data to assemble the plastid genome sequences. By using reference sequences, we filtered out 412,658–651,511 plastid reads from the raw reads for plastome assembly, which was 391 to 626 × coverage of the plastid genome of Ranunculeae. When assembling, we successfully bridged gaps by our previous method [17], and those gaps and IR/SC boundaries were confirmed by PCR amplification. All the newly assembled plastome sequences were deposited in the public online database GenBank under accession numbers from OR625572 to OR625582 (Table 1).

**Figure 1:**
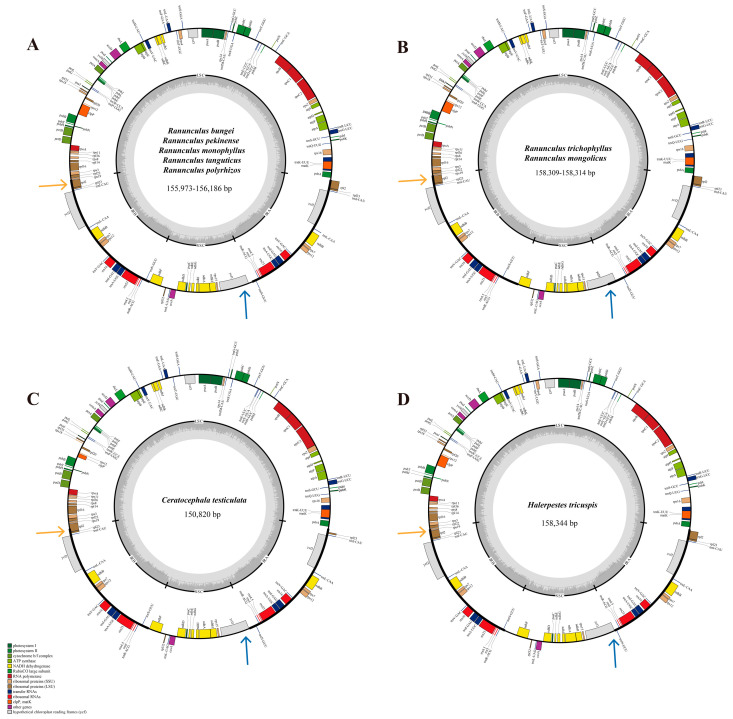
Gene maps of the newly sequenced plastome sequences of *Ranunculus* using Organellar Genome DRAW (**A**,**B**), *Ceratocephala* (**C**), and *Halerpestes* (**D**). For each circle, bold lines on the outer circle show the IR regions, while unbold lines indicate LSC and SSC regions. The inner track shows the G + C content. Genes transcribed in a clockwise direction are located on the outside of circle, while genes transcribed in a counterclockwise direction are on the inside of the map. LSC: large single copy region; SSC: small single copy region; IR: inverted repeat region. Arrows point the different IR-SC boundaries. Yellow and blue arrows indicate different changes at the same location in each of the four gene maps.

The length of all the newly assembled plastome sequences of Ranunculeae ranged from 150,820 bp (*C. testiculata*) to 158,344 bp (*H. tricuspis*) with the overall GC content of 36.7 to 37.4% (Figure 1; Supplementary Table S1). Within the genus *Ranunculus*, the length of plastome sequences ranged from 155,973 bp (*R. monophyllus*) to 158,314 bp (*R. trichophyllus*), with the overall GC content of 36.7 to 36.8%. In Ranunculeae samples, all the plastome sequences contained a LSC (83,575–86,441 bp), an SSC region (17,619–21,735 bp), and a pair of IRs (24,168–27,868 bp) regions and showed a typical structure in Angiosperms. A set of 112 genes were present in the plastomes of Ranunculeae samples, among which 78 are protein-coding genes, 30 are transfer RNAs, and 4 are ribosomal RNA genes (Table 2). A total of 16 (*Ceratocephala* samples) and 17 (other newly sequenced samples) genes were located in a single IR region. A total of 18 (in *Ranunculus* and *Halerpestes* samples) and 17 (in *Ceratocephala* samples) genes have introns (Supplementary Table S2). In *Ranunculus* and *Halerpestes* samples, the longest intron is in the *clp*P gene (1497 bp in *R. polyrhizos* −1562 bp in *H. tricuspis*), whereas in *Ceratocephala* samples, the longest intron is in the *ycf*3 gene (1442 bp).

### 3.2. Comparative Results of the Plastomes

**Figure 2:**
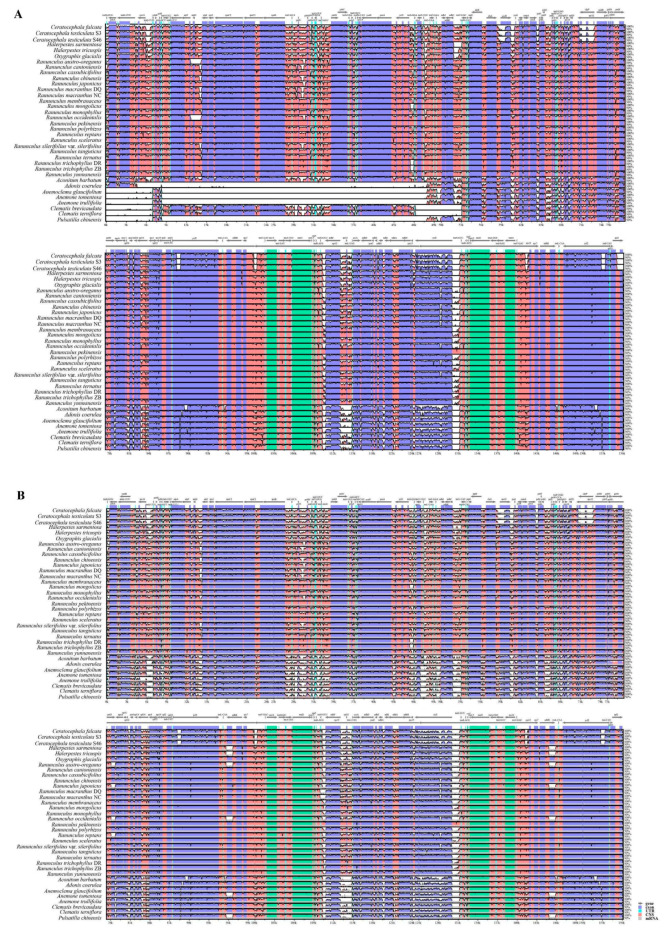
Multiple sequence alignments of Ranunculeae samples and its allies by mVISTA program. (**A**): alignment with LAGAN method, the white (empty) regions in the Anemoneae and Adonideae samples are the inverted and transposed regions; (**B**): alignment with shuffle LAGAN method. Blue regions show the coding regions, while green shows the rRNA regions, and pink shows the non-coding regions.

Multiple alignments using mVISTA were carried out for Ranunculeae samples to investigate plastid genome structural variations. Species with both normal and specific (in *Adonis* and tribe Anemoneae) plastome structures were also included. Two methods, LAGAN and Shuffle-LAGAN, were conducted and shown in Figure 2. When using the LAGAN method, Ranunculeae plastomes showed the same gene order as that of the *Aconitum* samples, but large empty (mismatch) regions were found in the LSC regions of *Adonis* and tribe Anemoneae samples due to gene inversion or gene translocation events.

**Figure 3:**
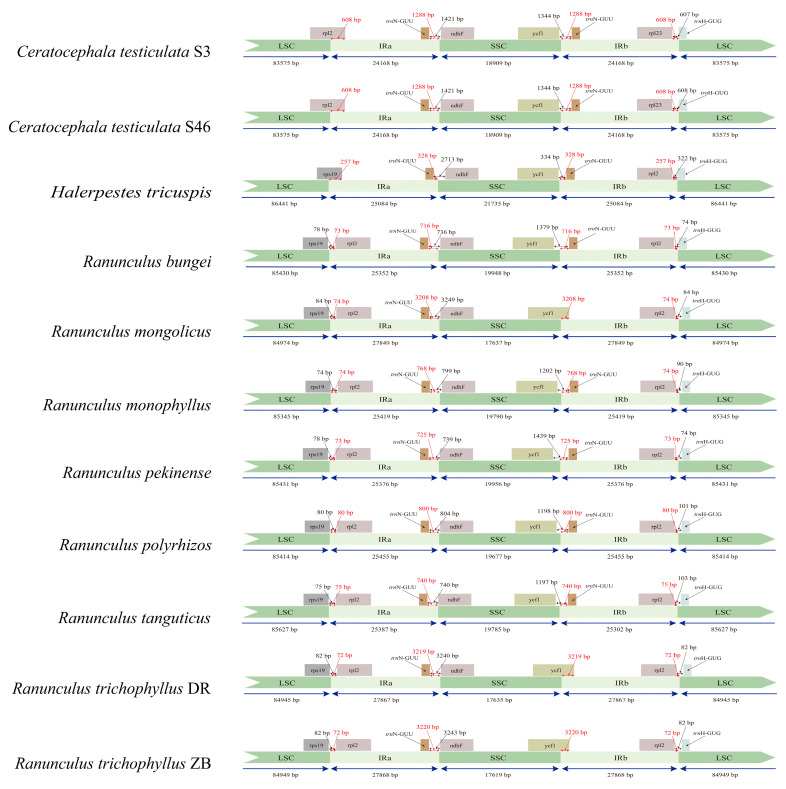
Detailed IR-SC boundaries of the newly sequenced samples. SC: single copy region; IR: inverted repeats.

Because the IR expansion/contraction may carry important phylogenetic information in Ranunculaceae [17], the IR/SC boundaries of the newly sequenced plastomes were compared with other outgroups in the family. The newly sequenced *Ranunculus* and *Halerpestes* samples as well as the published *Oxygraphis* sample in Ranunculeae have 17 genes in their IR region, which is the same as many other genera in Ranunculaceae (such as *Aconitum* L., *Caltha*, L., *Coptis* Salisb, *Delphinium* L., and *Thalictrum* L.) and other angiosperm taxa such as *Amborella* Baill. and *Arabidopsis* Heynh. [17,34]. Therefore, this 17-gene IR region of *Ranunculus* and *Halerpestes* can be taken as the primitive type in Ranunculaceae [17]. Whereas the IR regions of *Ceratocephala* samples showed slight contraction with incomplete *rpl*2 genes on the LSC/IR borders compared to the *Ranunculus* and *Halerpestes* samples (Figure 3).

**Figure 4:**
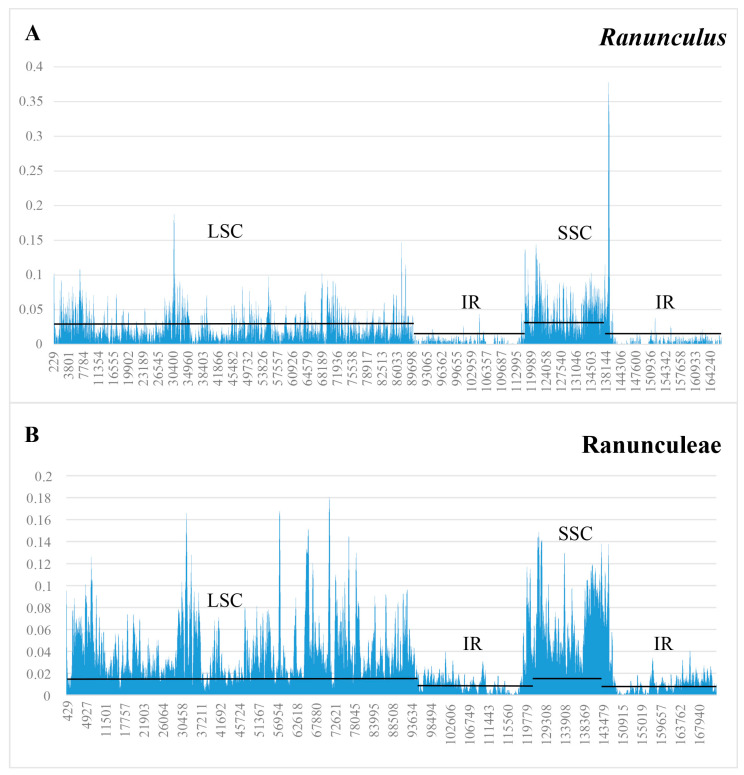
Graph of sliding window analysis showing plastome nucleotide variability (Pi) of *Ranunculus* (**A**) and Ranunculeae (**B**).

Nucleotide variability was assessed by sliding window analysis, and the results (Figure 4) showed that the IR region has a lower variability than the SC regions in *Ranunculus* samples. When taking all the Ranunculeae samples into consideration, the trend of lower nucleotide variability in the IR region is also obvious. In *Ranunculus* samples, our result discovered extremely high variations at the border of the IR/SSC regions.

### 3.3. Synonymous Codon Usage

**Figure 5:**
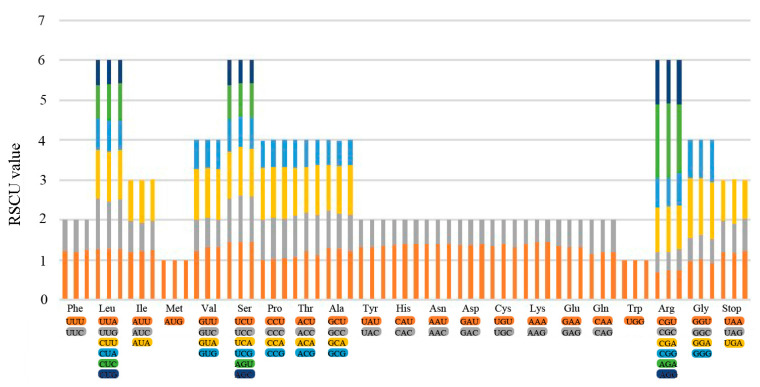
The values of relative synonymous codon usage for the 20 amino acids and stop codons in the plastomes of the newly sequenced samples.

This study calculated the relative synonymous codon usage (RSCU) for the newly assembled plastome sequences using all the protein-coding genes. We presented results of amino acid frequency and putative RNA editing sites in Figure 5 and Supplementary Tables S2 and S3. We detected 95 putative RNA editing sites in the 24 protein-coding genes of *Ceratocephala*, 92 sites in 27 protein-coding genes of *Halerpestes*, 93 sites in 27 protein-coding genes of *R. bungei* and *R. pekinensis*, and 91 sites in 27 protein-coding genes of the other five *Ranunculus* species. In the *Ranunculus* samples, *ndh*F has the most RNA editing sites (10 and 11 sites), and the second was *mat*K (9 sites). In the *Ceratocephala* samples, *rpo*C2 gene has the most RNA editing sites (12 sites), and the second was *ndh*F (11 sites). For the *Halerpestes* samples, both *rpo*C2 and *ndh*F genes have the most RNA editing sites (10 sites), and then was *ndh*B gene (8 sites).

The substitution from serine to leucine was tested to be the most common type (30.1%) in *R. bungei* and *R. pekinensis*, followed by serine to phenylalanine (15.1%), whereas in the other *Ranunculus* species, serine to leucine was the most common one (29.7%), followed by threonine to isoleucine (14.3%). In *Ceratocephala*, substitution from serine to leucine accounted for 26.3% of the editing sites, and from serine to phenylalanine was 13.7%. In *Halerpestes*, 31.5% of editing sites substituted from serine to leucine, and 15.2% from serine to phenylalanine, and among all its RNA editing sites, 23 substitutions appeared at the first nucleotide positions while 71 substitutions occurred at the second nucleotide position. Plastomes of the other two genera showed similar results in the substitution site on the codon positions (Supplementary Table S2).

### 3.4. SSR, Repetitive Sequences and Positive Selection Analysis

Rich SSRs including mononucleotide to hexonucleotide repeats were detected ranging from 47 to 70 in the newly sequenced plastomes (Supplementary Table S4). Among all the tested species, *C. testiculata* has the fewest SSRs, whereas *H. tricuspis* has the most. The most common SSR is mononucleotide repeat (A/T) among the nine species. For the tested species, the least proportion (53.2%) of the mononucleotide repeats was in *C. testiculata*, whereas the highest proportion (70.0%) was in *H. tricuspis*. The rare mononucleotide repeat (G/C) was only found in *R. mongolicus*, *R. monophyllus,* and *R. trichophyllus*. The second most common SSR is dinucleotide repeat (AT/TA) with six, eight, and nine replicates, respectively. The third most common SSR is tetranucleotide repeat (AATG/TGAA) with six, seven, nine, and ten replicates, respectively, and its total number was slightly smaller than the dinucleotide repeats. The fourth most common SSR is trinucleotide repeat (AAT/TTA), whereas pentanucleotide repeats were present in all the tested samples but *R. mongolicus*, and hexanucleotide repeats were only present in the plastomes of *H. tricuspis*, *R. bungei*, and *R. pekinense*. Within the newly sequenced plastomes, the largest proportion of SSR loci were found in IGS, followed by CDS and Intron. Within the plastid genome circle, SSRs are most common in the LSC region, followed by SSC, and the least in IR regions (Supplementary Table S4).

**Figure 6:**
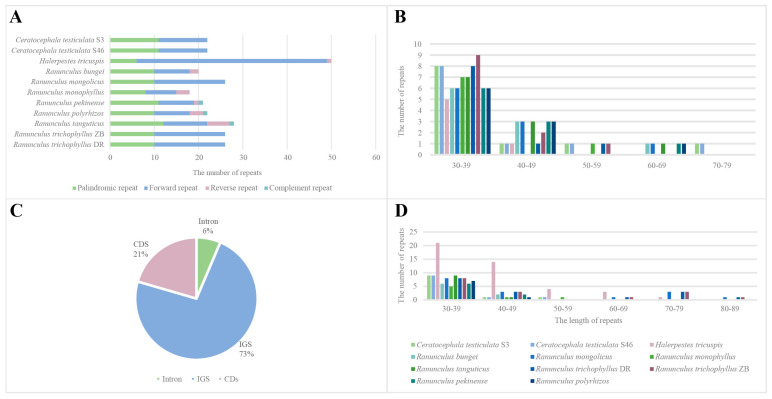
Graphs of repeated sequence analyses for the newly assembled plastomes. (**A**) Histogram of four repeat type numbers; (**B**) Histogram of palindromic repeats by length; (**C**) Pie chart showing proportion of repeats in different locations; (**D**) Histogram of forward repeats by length.

The eleven newly sequenced plastomes had a total of 281 direct, reverse, palindromic, and complement repeats (Figure 6), which may serve as potential molecular markers for further population genetic studies. Direct repeat was tested to be the most common repeat type, which accounted for 54.8% of the total repeats. It was followed by palindromic repeat (38.8%), reverse repeat (5.3%), and complement repeat (1.1%). The only three complement repeats were found in *R. pekinense*, *R. polyrhizos*, *R. tanguticus*, respectively. Repeats were usually short with 30–49 bp in length. We also found several longer direct and reverse repeats up to 82 bp in some *Ranunculus* samples. The largest proportion of repeats was found in the IGS region (73%), followed by CDS (21%) and Intron (6%) (Figure 6).

Positive selection of 67 CDS was tested for all the available Ranunculeae samples and its close allies. The likelihood ratio analysis showed that *p*-values of most genes were >0.05 (insignificant), except that *atp*B, *ndh*C, *ndh*G, *ndh*J, *psa*C, *rps*2, *rps*15, *ycf*2 (*p* < 0.05). Furthermore, the nonsynonymous/synonymous rate ratio (ω = dN/dS) of only one gene, *acc*D, is >1, but its *p*-value is >0.05. However, the BEB test showed that *acc*D, *atp*F, *ccs*A, *ndh*F, *pet*D, *rbc*L, *rpo*A, *rpo*C2 and *ycf*2 have high posterior probability values (≥0.9) (Supplementary Table S5). Previous studies considered that a coding region with a high posterior probability value of the BEB analysis can be taken as a positive selection gene [35]. Under this measure, nine genes, *acc*D, *atp*F, *ccs*A, *ndh*F, *pet*D, *rbc*L, *rpo*A, *rpo*C2, and *ycf*2 can be considered as positive selection genes.

### 3.5. Partitioning and Phylogenetic Reconstruction Results

We tested the complete plastome dataset by using six data partitioning strategies. The results showed that those six partitioning treatments obtained quite different results, indicating that different partitioning methods may greatly affect phylogenetic reconstruction. It showed that the partitioning strategy by LSC, SSC, and IRs obtained the best results (Table 3). According to this result, this partitioning strategy was applied for CDS, IGS, introns, and complete datasets, and no partitioning strategy was applied for the rest of the datasets. The GTR model for each partition of CDS, IGS, introns, and complete datasets was applied for BI analysis, while the GTR + I + G model was used for the rest of the datasets for both ML and BI analyses according to our partition results.

**Figure 7:**
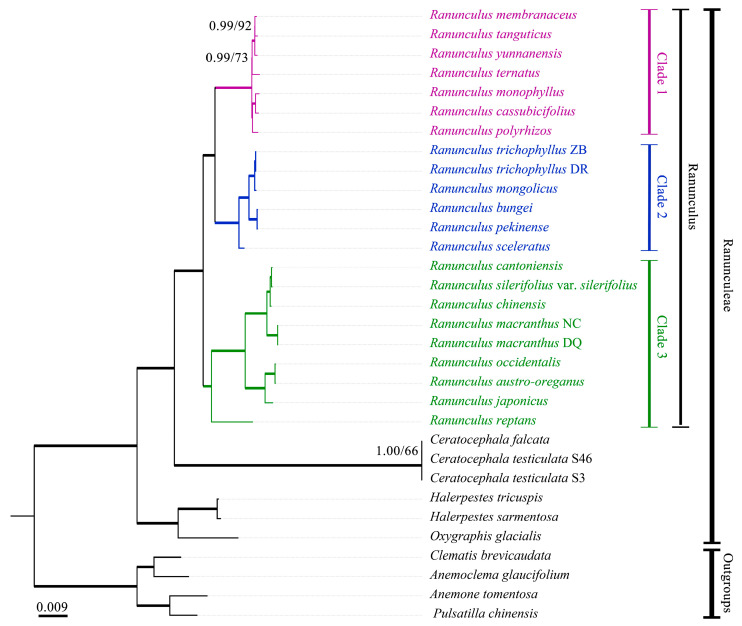
The Bayesian phylogenetic tree of all the currently available Ranunculaceae samples inferred from the complete plastome data. Numbers on nodes indicate maximum likelihood (ML) bootstrap values/posterior probability (PP) values. Bold branches show the fully supported clades with the ML bootstrap values =100 and PP values = 1.

Phylogenies reconstructed by both complete and separate datasets and both methods (ML and BI) were generally the same, especially in strongly supported clades (Figure 7, Supplementary Figure S2). The complete plastome dataset generated the most robustly resolved phylogeny of Ranunculeae. For this reason, our discussion was based on the phylogenetic framework inferred from the complete plastome dataset. All four tested genera of the tribe Ranunculeae were strongly supported. Two genera *Halerpestes* and *Oxygraphis* were sister groups and formed a clade sister to another clade including *Ranunculus* and *Ceratocephala*. Three major clades were resolved in the largest genera *Ranunculus*. Clade 1 includes species mainly from sect. *Auricomus* (Spach) Schur [15]. Clade 2 comprises aquatic species from sect. *Batrachium* DC. and a hydrophilus species *R. sceleratus* L from sect. Hecatonia. Clade 3 includes species mainly from sect. *Flammula* and sect. *Acris* Schur [10].

## 4. Discussion

Many previous studies have reported that most of the plastid genomes of Ranunculaceae have a set of 112 genes, and this is also the case in Ranunculeae species [17,19,35]. All our sequenced Ranunculeae samples also showed the same results as previous reports. Gene inversions and gene loss in Ranunculaceae plastomes were revealed more than 20 years ago. Johansson [41] studied *Adonis* species using the restriction site mapping method and found large inversions and gene loss presented in their plastid genomes. In recent years, structural variations including gene inversions, gene translocations, and IR variations have been explicitly reported in Ranunculaceae [17,19]. The plastome structural variations of tribe Anemoneae also have structural variations, but in comparison with *Adonis,* their variations have differed in many aspects. In genera *Anemone* s. l. and *Anemoclema*, there are three large inversions in the LSC region of their plastomes, while in *Clematis* there are two large inversions and one large transposition in the LSC region of the plastome [17]. In Ranunculeae species, gene orders of the plastome are the same as those of most other genera (such as *Aconitum*, *Thalictrum,* and so on), and no gene inversions and translocations are found.

The IR region normally has 17 genes, and this type of IR was considered to be primitive in Ranunculaceae [17]. However, IR expansion/contractions are also very common in Ranunculaceae. He et al. [17] reported that many genera (such as *Asteropyrum*, *Anemone*, *Anemoclema*, *Clematis*, *Dichocarpum*, *Hepatica*, *Hydrastis*, *Naravelia*, and *Pulsatilla*) in Ranunculaceae have expanded IR regions, whereas only two genera, *Helleborus* and *Ceratocephala*, were found to have slightly contracted IR regions. Up to 27 genes in *Asteropyrum peltatum* were found in the plastome of the family, and IR expansion/contractions may carry important phylogenetic information [17]. In tribe Ranunculeae, the majority of tested species have 17 genes in their IR regions except for *Ceratocephala*, which showed a little contraction in the IR regions (Supplementary Table S1). IR contraction is rare in Ranunculaceae, and only found in *Helleborus* and *Ceratocephala* [17]. In these two genera, *rpl*2 is not completely located in the IR region (Figure 3), and these two generic cases seemed to have no phylogenetic relationship in Ranunculaceae [17]. However, both species of *Ceratocephala* tested to have the same contracted IR regions, indicating that this IR contraction may be a synapomorphy in the genus *Ceratocephala* within the Ranunculeae clade. Our results showed that plastome structural variation is not characteristic of Ranunculeae, but IR expansion/contraction may have phylogenetic information.

Simple sequence repeats (SSRs) for microsatellites have been widely applied for population genetics and evolutionary studies of Angiosperm species [42]. However, the use of the plastid SSRs has not been fully developed in Ranunculaceae. Our results showed that 47 to 70 plastid SSRs are found in the 11 new samples (Supplementary Table S4), and pentanucleotide repeats are very common in the plastomes of Ranunculeae species. The rich plastid SSR diversity can provide opportunities for future population genetic studies on Ranunculeae species.

In Ranunculaceae, the tribe Ranunculeae is characterized by its ascending unitegmic ovules (except *Myosurus* which has pendent ovules), often smaller sepals and larger petals, and petals with one or more nectary glands near the base [10]. Some taxonomists included *Callianthemum* and *Adonis* into Ranunculeae [43,44]. However, this treatment was not supported by molecular phylogenetic analysis [45,46]. A large number of molecular phylogenetic studies of Ranunculeae have been published [11,12,13,47,48,49] which helped us understand the delimitation and generic relationship of this tribe. Based on molecular phylogeny and comprehensive sampling, 19 genera were recognized within the tribe Ranunculeae [11,12,13]. However, most of them were based on small numbers of DNA regions (nrITS and plastid DNA fragments), and the phylogenetic relationship within the tribe was still not robustly resolved. In this study, the generic relationship of the tribe inferred from the complete plastome data was congruent with previous studies and more robustly resolved (Figure 7 and Supplementary Figure S2), therefore demonstrating that plastome data may provide the opportunity for the reconstruction of generic phylogeny of Ranunculeae in the future with comprehensive sampling. Our current sampling covered all the generic representatives in China. The results showed the aquatic sect. *Batrachium* should be included in *Ranunculus* but as a distinct genus. Generic statuses of *Ceratocephala*, *Halerpestes*, and *Oxygraphis* can be kept.

*Ranunculus* is the largest genus in both Ranunculeae and Ranunculaceae with about 650 species around the world [10]. Taxonomy of *Ranunculus* has been considered extremely difficult and there are considerable differences among different classifications [9,15,50,51,52,53]. For this reason, this genus also attracted great attention in its phylogeny using molecular markers [47,54,55,56,57,58,59]. Based on a comprehensive sampling and nrITS and three plastid DNA regions, Emadzade et al. [57] resolved nine major clades in *Ranunculus*. Although we combined all the available complete plastome sequences of *Ranunculus* from GenBank, the sampling is still limited. Three major clades were robustly resolved by our plastome data. Clade 1 (Figure 6) corresponded to clade IV of Emadzade et al. [57] which included species of sect. *Auricomus*. Clade 1 also included *R. ternatus* from the sect. *Tuberifer* whose phylogenetic position has never been tested. Sect. *Tuberifer* is characterized by its tuberous roots. Wang [60] considered that *R. ternatus* may be closely related to sect. *Auricomus* and this prediction was supported by our phylogenetic analysis. Clade 2 included an aquatic sect. *Batrachium* and sect. *Hectonia* in wetland, and well corresponded to cluster III of Emadzade et al. [57]. The monophyly of cluster III in Emadzade et al. [57] was not supported in their study. However, clade 2 is fully supported showing the advantage of using the plastome data for phylogenetic reconstruction over the small number of DNA regions by Sanger sequencing. Clade 3 was also fully supported. In this clade, the first diverged *R. reptans* was in clade V of Emadzade et al. [57], and the other two subclades (*R. japonicus*–*R. occidentalis*) and (*R. cantoniensis*–*R. chinensis*) correspond with clade VI and Clade VIII of Emadzade et al. [57], respectively. Phylogenetic position of *R. macranthus* has never been inferred, and this species is also nested in clade 3. In general, the phylogenetic relationship within *Ranunculus* inferred by the complete plastome sequences was fully congruent with previous molecular studies and showed advantages of high resolution. Plastid phylogenomic analysis is needed for future studies with a comprehensive sampling.

## 5. Conclusions

The complete plastomes of eleven samples representing nine species of tribe Ranunculeae were de novo assembled using a next-generation sequencing method. The plastome sequences from all the Ranunculaceae samples and their allies were compared in various aspects including gene content, nucleotide variability, codon usage, RNA editing sites, simple sequence repeats, and positive selection sites through bioinformatic analyses. The phylogeny of Ranunculeae was reconstructed for the complete and separated datasets using both ML and BI methods to infer generic and specific relationships within the tribe. Our results showed that the majority of the Ranunculeae genera and species have the most common plastid genome type, which is widely shared in the family [17], and there are potential values of the plastome sequences for reconstructing the phylogeny of both the tribe and the genus *Ranunculus* in future studies.

## Figures and Tables

**Table 1:** Sample information pertaining to the present study.

Tribe	Species	Collecting Site	Voucher Number	GenBank No.
Ranunculeae	Ceratocephala testiculata *	Altay, Xinjiang, China	L. Xie 2016S3 (BJFC)	OR625574
Ranunculeae	Ce. testiculata *	Altay, Xinjiang, China	L. Xie 2016S46 (BJFC)	OR625575
Ranunculeae	Ranunculus monophyllus *	Altay, Xinjiang, China	L. Xie 2016S2 (BJFC)	OR625578
Ranunculeae	R. polyrhizos *	Altay, Xinjiang, China	L. Xie 2016S47 (BJFC)	OR625579
Ranunculeae	R. tanguticus *	Daocheng, Sichuan, China	W.H. Li WH072 (BJFC)	OR625580
Ranunculeae	R. mongolicus *	Qinghe, Xinjiang, China	C. Shang et al. I-4186 (BJFC)	OR625576
Ranunculeae	R. trichophyllus *	Tingri, Xizang, China	W.H. Li DR008 (BJFC)	OR625577
Ranunculeae	R. bungei *	Xinglong, Hebei, China	L. Xie et al. PL002 (BJFC)	OR625572
Ranunculeae	R. trichophyllus *	Xiangrila, Yunnan, China	L. Xie et al. T-20220808 016 (BJFC)	OR625582
Ranunculeae	R. pekinense *	Yanqing, Beijing, China	L. Xie and Y.K. Luo 20200916001 (BJFC)	OR625573
Ranunculeae	Halerpestes tricuspis *	Zhongba, Xizang, China	W.H. Li ZB006 (BJFC)	OR625581
Anemoneae	*Anemoclema glaucifolium*			MH205609
Anemoneae	*A. tomentosa*			NC_039451
Anemoneae	*Pulsatilla chinensis*			NC_039452
Anemoneae	*A. trullifolia*			MH205608
Anemoneae	*Clematis brevicaudata*			MT796620
Anemoneae	*Cl. terniflora*			KJ956785
Adonideae	*Adonis coerulea*			MK253469
Delphinieae	*Aconitum barbatum*			MK253470
Ranunculeae	*Ce. falcata*			MK253464
Ranunculeae	*H. sarmentosa*			MK253457
Ranunculeae	*Oxygraphis glacialis*			MK253453
Ranunculeae	*R. austro-oreganus*			KX639503
Ranunculeae	*R. cantoniensis*			NC_045920
Ranunculeae	*R. cassubicifolius*			OP250948
Ranunculeae	*R. chinensis*			ON500677
Ranunculeae	*R. japonicus*			MZ169045
Ranunculeae	*R. macranthus*			NC_008796
Ranunculeae	*R. macranthus*			DQ359689
Ranunculeae	*R. membranaceus*			NC_065303
Ranunculeae	*R. occidentalis*			NC_031651
Ranunculeae	*R. reptans*			NC_036977
Ranunculeae	*R. sceleratus*			MK253452
Ranunculeae	*R. silerifolius*			ON462450
Ranunculeae	*R. ternatus*			OQ943173
Ranunculeae	*R. yunnanensis*			MZ703201

* newly sequenced in this study.

**Table 2:** Genes present in the plastid genomes of the 11 newly sequenced Ranunculeae samples.

Gene Type	Gene Name				
Ribosomal RNA genes	16S rRNA	23S rRNA	4.5S rRNA	5S rRNA	
Transfer RNA genes	*trn*A-UGC gene	*trn*C-GCA gene	*trn*D-GUC gene	*trn*E-UUC gene	*trn*F-GAA gene
	*trn*fM-CAU gene	*trn*G-GCC gene	*trn*G-UCC gene	*trn*H-GUG gene	*trn*I-CAU gene
	*trn*I-GAU gene	*trn*K-UUU gene	*trn*L-CAA gene	*trn*L-UAA gene	*trn*L-UAG gene
	*trn*M-CAU gene	*trn*N-GUU gene	*trn*P-UGG gene	*trn*Q-UUG gene	*trn*R-ACG gene
	*trn*R-UCU gene	*trn*S-GCU gene	*trn*S-GGA gene	*trn*S-UGA gene	*trn*T-GGU gene
	*trn*T-UGU gene	*trn*V-UAC gene	*trn*V-GAC gene	*trn*W-CCA gene	*trn*Y-GUA gene
Small subunit of the ribosome	*rps*2 gene	*rps*3 gene	*rps*4 gene	*rps*7 gene	*rps*8 gene
	*rps*11 gene	*rps*12 gene	*rps*14 gene	*rps*15 gene	*rps*16 gene
	*rps*18 gene	*rps*19 gene			
The large subunit of the ribosome	*rpl*2 gene	*rpl*14 gene	*rpl*16 gene	*rpl*20 gene	*rpl*22 gene
	*rpl*23 gene	*rpl*32 gene	*rpl*33 gene	*rpl*36 gene	
RNA polymerase subunits	*rpo*A gene	*rpo*B gene	*rpo*C1 gene	*rpo*C2 gene	
NADH dehydrogenase	*ndh*A gene	*ndh*B gene	*ndh*C gene	*ndh*D gene	*ndh*E gene
	*ndh*F gene	*ndh*G gene	*ndh*H gene	*ndh*I gene	*ndh*J gene
	*ndh*K gene				
Photosystem I	*psa*A gene	*psa*B gene	*psa*C gene	*psa*I gene	*psa*J gene
Cytochrome b/f complex	*pet*A gene	*pet*B gene	*pet*D gene	*pet*G gene	*pet*L gene
	*pet*N gene				
ATP synthase	*atp*A gene	*atp*B gene	*atp*E gene	*atp*F gene	*atp*H gene
	*atp*I gene				
Large subunit of rubisco	*rbc*L gene				
Maturase	*mat*K gene				
Protease	*clp*P gene				
Envelope membrane protein	*cem*A gene				
Subunit of acetyl-CoA-carboxylase	accD gene				
Photosystem II	*psb*A gene	*psb*B gene	*psb*C gene	*psb*D gene	*psb*E gene
	*psb*F gene	*psb*H gene	*psb*I gene	*psb*J gene	*psb*K gene
	*psb*L gene	*psb*M gene	*psb*N gene	*psb*T gene	*psb*Z gene
Copper chaperone for superoxide dismutase	*ccs*A gene				
Conserved open reading frames	*Ycf* 1,2,3,4				

**Table 3:** Partitioning strategy tests for the complete plastid genome dataset using PartitionFinder.

Dataset	Partitioning Strategy	Parameters	Subsets	ln L	BIC
Complete Plastome Dataset	No partition	63	1	−492,025.19	984,804.90
	Coding and non-coding	74	2	−600,890.34	1,202,691.59
	LSC, SSC, IRs	85	3	−489,387.16	979,792.32
	By gene	162	11	−710,387.28	1,422,765.77
	By gene and codon position	219	17	−708,937.30	1,420,566.42
	By the third codon position	84	3	−802,253.60	1,605,550.34

## Data Availability

The genome sequence data that support the findings of this study are openly available from NCBI GenBank (https://www.ncbi.nlm.nih.gov, accessed on 30 September 2023) under accession numbers from OR625572 to OR625582. The aligned datasets are available on Zenodo, with the identifier https://doi.org/10.5281/zenodo.10012320, accessed on 20 September 2023.

## Supplementary Materials

The following supporting information can be downloaded at: https://www.mdpi.com/article/10.3390/genes14122140/s1, Figure S1: Detailed IR-SC boundaries of all the tested samples. SC: single copy region; IR: inverted repeats. Figure S2: The Bayesian phylogenetic tree of all the currently available Ranunculaceae samples inferred from the separated data. Numbers on nodes indicate maximum likelihood (ML) bootstrap values/posterior probability (PP) values. Bold branches show the fully supported clades with the ML bootstrap values = 100 and PP values = 1. Table S1: Major plastid genome features of the 11 newly sequenced samples of Ranunculeae. Table S2: Prediction of RNA editing by the PREP-cp program. Table S3: Base composition in the plastid genome of each sample. Table S4: Information of simple sequence repeats (SSRs) for the 11 newly sequenced Ranunculeae samples. Table S5: The potential positive selection test based on the branch-site model.

## Abbreviations

SC	Single copy region
LSC	Large single copy region
SSC	Small single copy region
IR	Inverted repeat region
CDS	Coding regions
IGS	Intergenic spacer regions
dN	Nonsynonymous substitution
dS	Synonymous substitution
Pi	Nucleotide variability
BEB	Bayes Empirical Bayes
PP	Posterior probability values
ML	Maximum likelihood
BI	Bayesian inference
BIC	Bayesian information criterion
SSRs	Simple sequence repeats
RSCU	Relative synonymous codon usage
NGS	Next-generation sequencing
MISA	MIcroSAtellite
