# Supplementary material for: Complete Plastid Genomes of Nine Species of Ranunculeae (Ranunculaceae) and Their Phylogenetic Inferences
Source: Genes (Basel). 2023 Nov 27;14(12):2140. doi: 10.3390/genes14122140 (PMC10742492; doi:10.3390/genes14122140)
Supplement: Supplementary file 1 [file genes-14-02140-s001.zip › Table S1.pdf]

Table S1. Major chloroplast genome features of the 11 newly sequenced samples of Ranunculeae.

Features	*Ranunculus bungei*	*R. pekinense*	*R. trichophyllus* DR	*R. monophyllus*	*R. tanguticus*	*R. polyrhizos*
Genome size	156082	156139	158314	155973	156186	156000
Length of LSC	85430	85431	84945	85345	85627	85414
Length of SSC	19948	19956	17635	19790	19785	19677
Length of IR	25352	25376	27857	25419	25387	25455
Total G+C content (%)	36.70%	36.70%	36.80%	36.80%	36.80%	36.80%
Total number of genes	112	112	112	112	112	112
Protein encoding	78	78	78	78	78	78
tRNA	30	30	30	30	30	30
rRNA	4	4	4	4	4	4
Duplicated in IRs	17	17	17	17	17	17
Genes with introns	18	18	18	18	18	18
Features	*R. mongolicus*	*R. trichophyllus* ZB	*Ceratocephala testiculata S3*	*C. testiculata* S46	*Halerpestes tricuspis*	
Genome size	158309	158304	150820	150820	158344	
Length of LSC	84974	84949	83575	83575	86441	
Length of SSC	17637	17619	18909	18909	21735	
Length of IR	27849	27868	24168	24168	25084	
Total G+C content (%)	36.80%	36.80%	37.40%	37.40%	36.80%	
Total number of genes	112	112	112	112	112	
Protein encoding	78	78	78	78	78	
tRNA	30	30	30	30	30	
rRNA	4	4	4	4	4	
Duplicated in IRs	17	17	16	16	17	
Genes with introns	18	18	17	17	18	
