# Supplementary material for: Complete Plastid Genomes of Nine Species of Ranunculeae (Ranunculaceae) and Their Phylogenetic Inferences
Source: Genes (Basel). 2023 Nov 27;14(12):2140. doi: 10.3390/genes14122140 (PMC10742492; doi:10.3390/genes14122140)
Supplement: Supplementary file 1 [file genes-14-02140-s001.zip › Table S2.pdf]

Table S2. Prediction of RNA editing by the PREP-cp program.

***a. Ranunculus* 1(*Ranunculus bungei* and *Ranunculus pekinensis*)**

Gene	Nucleotide position	Triplet position	Bases	Codon change	Amino acid change
*acc*D	178	1	C-T	CAT-TAT	H-Y
	403	1	C-T	CAC- TAC	H-Y
	1172	2	C-T	TCA-TTA	S-L
*atp*B	1184	2	C-T	TCA-TTA	S-L
*atp*F	92	2	C-T	CCA-CTA	P-L
*atp*I	428	2	C-T	CCT-CTT	P-L
	629	2	C-T	TCA-TTA	S-L
*ccs*A	515	2	C-T	TCT-TTT	S-F
	548	2	C-T	CCA-CTA	P-L
	559	1	C-T	CTT-TTT	L-F
	625	1	C-T	CAC-TAC	H-Y
	956	2	C-T	TCT-TTT	S-F
*clp*P	79	1	C-T	CAT-TAT	H-Y
	556	1	C-T	CAT-TAT	H-Y
*mat*K	401	2	C-T	ACA-ATA	T-I
	439	1	C-T	CAT-TAT	H-Y
	578	2	C-T	ACT-ATT	T-I
	652	1	C-T	CAT-TAT	H-Y
	743	2	C-T	ACA-ATA	T-I
	1034	2	C-T	TCG-TTG	S-L
	1196	2	C-T	TCA-TTA	S-L
	1198	1	C-T	CCG-TCG	P-S
	1247	2	C-T	TCT-TTT	S-F
*ndh*B	467	2	C-T	CCA-CTA	P-L
	586	1	C-T	CAT-TAT	H-Y
	611	2	C-T	TCA-TTA	S-L
	746	2	C-T	TCT-TTT	S-F
	830	2	C-T	TCA-TTA	S-L
	1112	2	C-T	TCA-TTA	S-L
	1255	1	C-T	CAT-TAT	H-Y
	1481	2	C-T	CCG-CTG	P-L
*ndh*D	29	2	C-T	ACG-ATG	T-M
	626	2	C-T	TCA-TTA	S-L
	637	1	C-T	CCC-TCC	P-S
	905	2	C-T	TCA-TTA	S-L
	1211	2	C-T	GCT-GTT	A-V
	1292	2	C-T	ACG-ATG	T-M
	1325	2	C-T	TCA-TTA	S-L
	1502	2	C-T	GCG-GTG	A-V
*ndh*F	79	1	C-T	CTC- TTC	L-F

	199	1	C-T	CCT-TCT	P-S
	365	2	C-T	TCG-TTG	S-L
	586	1	C-T	CTT-TTT	L-F
	692	2	C-T	TCC- TTC	S-F
	1298	2	C-T	ACA-ATA	T-I
	1450	1	C-T	CCA-TCA	P-S
	1567	1	C-T	CTT-TTT	L-F
	1586	2	C-T	TCT-TTT	S-F
	1607	2	C-T	TCC-TTC	S-F
	2135	2	C-T	ACT-ATT	T-I
*ndh*G	137	2	C-T	ACA-ATA	T-I
	152	2	C-T	ACT- ATT	T-I
	155	2	C-T	CCA-CTA	P-L
	166	1	C-T	CAT-TAT	H-Y
	314	2	C-T	ACA-ATA	T-I
*pet*B	611	2	C-T	CCA-CTA	P-L
*psa*B	1256	2	C-T	ACA-ATA	T-I
*psa*I	80	2	C-T	TCT-TTT	S-F
*psb*F	77	2	C-T	TCT-TTT	S-F
*rpl*2	2	2	C-T	ACG-ATG	T-M
*rpl*20	224	2	C-T	TCC-TTC	S-F
	269	2	C-T	TCG-TTG	S-L
	308	2	C-T	TCA-TTA	S-L
*rpl*23	71	2	C-T	TCT-TTT	S-F
*rpo*A	368	2	C-T	TCA-TTA	S-L
	833	2	C-T	TCA-TTA	S-L
*rpo*B	247	1	C-T	CCC-TCC	P-S
	473	2	C-T	TCA-TTA	S-L
	548	2	C-T	CCA-CTA	P-L
	551	2	C-T	TCA-TTA	S-L
	566	2	C-T	TCA-TTA	S-L
	2426	2	C-T	TCA-TTA	S-L
*rpo*C1	488	2	C-T	TCA-TTA	S-L
	787	1	C-T	CGG-TGG	R-W
*rpo*C2	668	2	C-T	ACC-ATC	T-I
	1585	1,2	C-T	CCT-TTT	P-F
	1586	1,2	C-T	CCT-TTT	P-F
	2348	2	C-T	ACA-ATA	T-I
	2390	2	C-T	CCT- CTT	P-L
	2810	2	C-T	ACT-ATT	T-I
	3041	2	C-T	TCC-TTC	S-F
	3713	2	C-T	TCA-TTA	S-L
*rps*2	248	2	C-T	TCA-TTA	S-L

	314	2	C-T	TCA-TTA	S-L
	668	2	C-T	TCC-TTC	S-F
*rps*8	182	2	C-T	TCA-TTA	S-L
	271	1	C-T	CAT-TAT	H-Y
*rps*14	80	2	C-T	TCA-TTA	S-L
	149	2	C-T	CCA-CTA	P-L
	194	2	C-T	TCG-TTG	S-L
*rps*16	143	2	C-T	TCA-TTA	S-L
*ycf*3	185	2	C-T	ACG-ATG	T-M
	407	2	C-T	TCC-TTC	S-F

***b. Ranunculus* 2(others)**

Gene	Nucleotide position	Triplet position	Bases	Codon change	Amino acid change
*acc*D	13	1	C-T	CGG-TGG	R-W
	178	1	C-T	CAT-TAT	H-Y
	403	1	C-T	CAC-TAC	H-Y
	1172	2	C-T	TCA-TTA	S-L
*atp*B	1184	2	C-T	TCA-TTA	S-L
*atp*F	92	2	C-T	CCA-CTA	P-L
*atp*I	428	2	C-T	CCT-CTT	P-L
	629	2	C-T	TCA-TTA	S-L
*ccs*A	524	2	C-T	CCG-CTG	P-L
	553	1	C-T	CTT-TTT	L-F
	634	1	C-T	CAC-TAC	H-Y
	965	2	C-T	TCT-TTT	S-F
*clp*P	79	1	C-T	CAT-TAT	H-Y
	556	1	C-T	CAT-TAT	H-Y
*mat*K	392	2	C-T	ACA-ATA	T-I
	569	2	C-T	ACT-ATT	T-I
	643	1	C-T	CAT-TAT	H-Y
	734	2	C-T	ACA-ATA	T-I
	1025	2	C-T	TCG-TTG	S-L
	1187	2	C-T	TCA-TTA	S-L
	1189	1	C-T	CCA-TCA	P-S
	1238	2	C-T	TCT-TTT	S-F
	1423	1	C-T	CTC-TTC	L-F
*ndh*B	467	2	C-T	CCA-CTA	P-L
	586	1	C-T	CAT-TAT	H-Y
	611	2	C-T	TCA-TTA	S-L
	746	2	C-T	TCT-TTT	S-F
	830	2	C-T	TCA-TTA	S-L
	1112	2	C-T	TCA-TTA	S-L

	1255	1	C-T	CAT-TAT	H-Y
	1481	2	C-T	CCG-CTG	P-L
*ndh*D	29	2	C-T	ACG-ATG	T-M
	626	2	C-T	TCA-TTA	S-L
	637	1	C-T	CCC-TCC	P-S
	905	2	C-T	TCA-TTA	S-L
	1211	2	C-T	GCT-GTT	A-V
	1292	2	C-T	ACG-ATG	T-M
	1325	2	C-T	TCA-TTA	S-L
	1502	2	C-T	GCT-GTT	A-V
*ndh*F	79	1	C-T	CTC-TTC	L-F
	199	1	C-T	CCT-TCT	P-S
	586	1	C-T	CTT-TTT	L-F
	632	2	C-T	ACA-ATA	T-I
	692	2	C-T	TCT-TTT	S-F
	1172	2	C-T	GCG-GTG	A-V
	1298	2	C-T	ACA-ATA	T-I
	1564	1	C-T	CTC-TTC	L-F
	1604	2	C-T	TCC-TTC	S-F
	2132	2	C-T	ACT-ATT	T-I
*ndh*G	137	2	C-T	ACA-ATA	T-I
	152	2	C-T	ACT-ATT	T-I
	155	2	C-T	CCA-CTA	P-L
	314	2	C-T	ACA-ATA	T-I
*pet*B	611	2	C-T	CCA-CTA	P-L
*psa*B	1256	2	C-T	ACA-ATA	T-I
*psa*I	80	2	C-T	TCT-TTT	S-F
*psb*F	77	2	C-T	TCT-TTT	S-F
*rpl*2	2	2	C-T	ACG-ATG	T-M
*rpl*20	224	2	C-T	TCC-TTC	S-F
	269	2	C-T	TCG-TTG	S-L
	308	2	C-T	TCA-TTA	S-L
*rpl*23	71	2	C-T	TCT-TTT	S-F
*rpo*A	368	2	C-T	TCA-TTA	S-L
	833	2	C-T	TCA-TTA	S-L
*rpo*B	247	1	C-T	CCC-TCC	P-S
	473	2	C-T	TCA-TTA	S-L
	551	2	C-T	TCA-TTA	S-L
	566	2	C-T	TCG-TTG	S-L
	2426	2	C-T	TCA-TTA	S-L
*rpo*C1	488	2	C-T	TCA-TTA	S-L
	787	1	C-T	CGG-TGG	R-W
	1843	1	C-T	CCA-TCA	P-S

*rpo*C2	668	2	C-T	ACC-ATC	T-I
	1585	1,2	C-T	CCT-TTT	P-F
	1586	1,2	C-T	CCT-TTT	P-F
	2348	2	C-T	ACA-ATA	T-I
	2390	2	C-T	CCT-CTT	P-L
	2810	2	C-T	ACT-ATT	T-I
	3041	2	C-T	TCC-TTC	S-F
	3713	2	C-T	TCA-TTA	S-L
*rps*2	248	2	C-T	TCA-TTA	S-L
	314	2	C-T	TCA-TTA	S-L
	668	2	C-T	TCC-TTC	S-F
*rps*8	182	2	C-T	TCA-TTA	S-L
	271	1	C-T	CAT-TAT	H-Y
*rps*14	80	2	C-T	TCA-TTA	S-L
	149	2	C-T	CCA-CTA	P-L
	194	2	C-T	TCG-TTG	S-L
*rps*16	143	2	C-T	TCA-TTA	S-L
*ycf*3	185	2	C-T	ACG-ATG	T-M
	407	2	C-T	TCC-TTC	S-F

c. Ceratocephala

Gene	Nucleotide position	Triplet position	Bases	Codon change	Amino acid change
*acc*D	74	2	C-T	ACA-ATA	T-I
	403	1	C-T	CCA-TCA	P-S
	538	1	C-T	CAT-TAT	H-Y
	797	2	C-T	TCA-TTA	S-L
*atp*B	1184	2	C-T	TCA-TTA	S-L
*atp*F	92	2	C-T	CCA-CTA	P-L
*atp*I	428	2	C-T	CCT-CTT	P-L
	629	2	C-T	TCA-TTA	S-L
*ccs*A	515	2	C-T	TCT-TTT	S-F
	542	2	C-T	CCG-CTG	P-L
	553	1	C-T	CTT-TTT	L-F
	557	2	C-T	TCT-TTT	S-F
	637	1	C-T	CAT-TAT	H-Y
*mat*K	280	1	C-T	CCT-TCT	P-S
	398	2	C-T	ACA-ATA	T-I
	436	1	C-T	CAT-TAT	H-Y
	649	1	C-T	CAT-TAT	H-Y
	740	2	C-T	ACA-ATA	T-I
	1031	2	C-T	TCG-TTG	S-L
	1193	2	C-T	TCA-TTA	S-L

	1244	2	C-T	TCT-TTT	S-F
	1429	1	C-T	CTC-TTC	L-F
*ndh*B	467	2	C-T	CCA-CTA	P-L
	586	1	C-T	CAT-TAT	H-Y
	611	2	C-T	TCA-TTA	S-L
	746	2	C-T	TCT-TTT	S-F
	830	2	C-T	TCA-TTA	S-L
	1112	2	C-T	TCA-TTA	S-L
	1255	1	C-T	CAT-TAT	H-Y
	1481	2	C-T	CCA-CTA	P-L
*ndh*D	2	2	C-T	ACG-ATG	T-M
	599	2	C-T	TCA-TTA	S-L
	610	1	C-T	CCA-TCA	P-S
	620	2	C-T	GCA-GTA	A-V
	878	2	C-T	TCA-TTA	S-L
	1265	2	C-T	ACG-ATG	T-M
	1298	2	C-T	TCA-TTA	S-L
	1475	2	C-T	GCT-GTT	A-V
*ndh*F	79	1	C-T	CTC-TTC	L-F
	179	2	C-T	ACT-ATT	T-I
	199	1	C-T	CCT-TCT	P-S
	586	1	C-T	CTT-TTT	L-F
	692	2	C-T	TCT-TTT	S-F
	1172	2	C-T	GCA-GTA	A-V
	1298	2	C-T	ACA-ATA	T-I
	1478	2	C-T	TCA-TTA	S-L
	1525	1	C-T	CAT-TAT	H-Y
	1558	1	C-T	CTT-TTT	L-F
	1844	2	C-T	TCT-TTT	S-F
*ndh*G	137	2	C-T	ACA-ATA	T-I
	155	2	C-T	CCA-CTA	P-L
	166	1	C-T	CAT-TAT	H-Y
	314	2	C-T	ACA-ATA	T-I
	347	2	C-T	CCA-CTA	P-L
*psa*I	80	2	C-T	TCT-TTT	S-F
*psb*F	77	2	C-T	TCT-TTT	S-F
*rpl*2	2	2	C-T	ACG-ATG	T-M
*rpl*20	131	2	C-T	GCT-GTT	A-V
	224	2	C-T	TCC-TTC	S-F
	344	2	C-T	ACA-ATA	T-I
*rpl*23	71	2	C-T	TCT-TTT	S-F
*rpo*A	368	2	C-T	TCA-TTA	S-L
	836	2	C-T	TCA-TTA	S-L

*rpo*B	247	1	C-T	CCC-TCC	P-S
	473	2	C-T	TCA-TTA	S-L
	548	2	C-T	CCA-CTA	P-L
	566	2	C-T	TCA-TTA	S-L
*rpo*C1	488	2	C-T	TCA-TTA	S-L
	1687	1	C-T	CAT-TAT	H-Y
	1843	1	C-T	CCA-TCA	P-S
*rpo*C2	668	2	C-T	ACC-ATC	T-I
	1559	2	C-T	GCG-GTG	A-V
	1585	1,2	C-T	CCT-TTT	P-F
	1586	1,2	C-T	CCT-TTT	P-F
	1774	1	C-T	CGT-TGT	R-C
	1973	2	C-T	ACG-ATG	T-M
	2348	2	C-T	ACA-ATA	T-I
	2390	1	C-T	CCC-CTC	P-L
	2810	2	C-T	ACT-ATT	T-I
	3041	2	C-T	TCC-TTC	S-F
	3680	2	C-T	TCG-TTG	S-L
	3713	2	C-T	TCA-TTA	S-L
*rps*2	473	2	C-T	TCA-TTA	S-L
	527	2	C-T	CCC-CTC	P-L
	539	2	C-T	TCA-TTA	S-L
	832	1	C-T	CCG-TCG	P-S
	893	2	C-T	TCC-TTC	S-F
*rps*8	182	2	C-T	TCA-TTA	S-L
	271	2	C-T	CAT-TAT	H-Y
*rps*14	80	2	C-T	TCA-TTA	S-L
	149	2	C-T	CCA-CTA	P-L
	194	2	C-T	TCG-TTG	S-L
*rps*16	143	2	C-T	TCA-TTA	S-L
*ycf*3	185	2	C-T	ACG-ATG	T-M
	407	2	C-T	TCC-TTC	S-F

d. Halerpestes

Gene	Nucleotide position	Triplet position	Bases	Codon change	Amino acid change
*acc*D	365	2	C-T	CCG-CTG	P-L
	1160	2	C-T	TCA-TTA	S-L
*atp*B	22	1	C-T	CCG-TCG	P-S
	1184	2	C-T	TCA-TTA	S-L
*atp*F	92	2	C-T	CCA-CTA	P-L
*atp*I	428	2	C-T	CCT-CTT	P-L
	629	2	C-T	TCA-TTA	S-L

*ccs*A	524	2	C-T	CCG-CTG	P-L
	553	1	C-T	CTT-TTT	L-F
	634	1	C-T	CAC-TAC	H-Y
	965	2	C-T	TCT-TTT	S-F
*clp*P	79	1	C-T	CAT-TAT	H-Y
	556	1	C-T	CAT-TAT	H-Y
*mat*K	439	1	C-T	CAT-TAT	H-Y
	652	1	C-T	CAT-TAT	H-Y
	743	2	C-T	ACA-ATA	T-I
	1034	2	C-T	TCA-TTA	S-L
	1196	2	C-T	TCA-TTA	S-L
	1247	2	C-T	TCT-TTT	S-F
*ndh*B	467	2	C-T	CCA-CTA	P-L
	586	1	C-T	CAT-TAT	H-Y
	611	2	C-T	TCA-TTA	S-L
	746	2	C-T	TCT- TTT	S-F
	830	2	C-T	TCA-TTA	S-L
	1112	2	C-T	TCA-TTA	S-L
	1255	1	C-T	CAT-TAT	H-Y
	1481	2	C-T	CCA-CTA	P-L
*ndh*D	2	2	C-T	ACG-ATG	T-M
	383	2	C-T	TCA-TTA	S-L
	599	2	C-T	TCA-TTA	S-L
	878	2	C-T	TCA-TTA	S-L
	1184	2	C-T	GCT-GTT	A-V
	1265	2	C-T	ACG-ATG	T-M
	1298	2	C-T	TCA-TTA	S-L
*ndh*F	79	1	C-T	CTC-TTC	L-F
	199	1	C-T	CCC-TCC	P-S
	211	1	C-T	CAT-TAT	H-Y
	392	2	C-T	TCT-TTT	S-F
	692	2	C-T	TCC-TTC	S-F
	854	2	C-T	ACA-ATA	T-I
	1172	2	C-T	GCG-GTG	A-V
	1490	2	C-T	TCA-TTA	S-L
	1607	2	C-T	TCC-TTC	S-F
	1940	2	C-T	ACT-ATT	T-I
*ndh*G	137	2	C-T	ACA-ATA	T-I
	148	1	C-T	CAT-TAT	H-Y
	155	2	C-T	TCA-TTA	S-L
	166	1	C-T	CAT-TAT	H-Y
	314	2	C-T	ACA-ATA	T-I
	347	2	C-T	CCA-CTA	P-L

*pet*B	611	2	C-T	CCA-CTA	P-L
*psa*B	1256	2	C-T	ACA-ATA	T-I
*psa*I	80	2	C-T	TCT-TTT	S-F
*psb*F	77	2	C-T	TCT-TTT	S-F
*rpl*2	2	2	C-T	ACG-ATG	T-M
*rpl*20	224	2	C-T	TCC-TTC	S-F
	308	2	C-T	TCA-TTA	S-L
*rpl*23	71	2	C-T	TCT-TTT	S-F
*rpo*A	368	2	C-T	TCA-TTA	S-L
	830	2	C-T	TCA-TTA	S-L
	985	1	C-T	CTC-TTC	L-F
*rpo*B	338	2	C-T	TCT-TTT	S-F
	473	2	C-T	TCA-TTA	S-L
	548	2	C-T	CCA-CTA	P-L
	551	2	C-T	TCA-TTA	S-L
	566	2	C-T	TCG-TTG	S-L
	2426	2	C-T	TCA-TTA	S-L
*rpo*C1	488	2	C-T	TCA-TTA	S-L
	617	2	C-T	CCG-CTG	P-L
	787	1	C-T	CGG-TGG	R-W
	1843	1	C-T	CCA-TCA	P-S
*rpo*C2	668	2	C-T	ACC-ATC	T-I
	1465	1	C-T	CTT-TTT	L-F
	1585	1,2	C-T	CCT-TTT	P-F
	1586	1,2	C-T	CCT-TTT	P-F
	1774	1	C-T	CGT-TGT	R-C
	2287	1	C-T	CGG-TGG	R-W
	2348	2	C-T	ACC-ATC	T-I
	2810	2	C-T	ACT-ATT	T-I
	3041	2	C-T	TCC-TTC	S-F
	3707	2	C-T	TCA-TTA	S-L
*rps*2	248	2	C-T	TCA-TTA	S-L
	314	2	C-T	TCA-TTA	S-L
	668	2	C-T	TCC-TTC	S-F
*rps*8	182	2	C-T	TCA-TTA	S-L
	271	1	C-T	CAT-TAT	H-Y
*rps*14	80	2	C-T	TCA-TTA	S-L
	149	2	C-T	CCA-CTA	P-L
	194	2	C-T	TCG-TTG	S-L
*rps*16	143	2	C-T	TCA-TTA	S-L
*ycf*3	185	2	C-T	ACG-ATG	T-M
	407	2	C-T	TCC-TTC	S-F

e. Summary

Genus / Amino acid change	*Ranunculus* 1		*Ranunculus* 2		*Ceratocephala*		*Halerpestes*	
	Numbers	Percentage	Numbers	Percentage	Numbers	Percentage	Numbers	Percentage
A-V	2	2.2%	3	3.3%	5	5.3%	2	2.2%
H-Y	11	11.8%	9	9.9%	10	10.5%	11	12.0%
L-F	4	4.3%	5	5.5%	5	5.3%	4	4.3%
P-F	2	2.2%	2	2.2%	2	2.1%	2	2.2%
P-L	10	10.8%	9	9.9%	11	11.6%	11	12.0%
P-S	5	5.4%	5	5.5%	7	7.4%	3	3.3%
R-C	-	-	-	-	1	1.1%	1	1.1%
R-W	1	1.1%	2	2.2%	-	-	2	2.2%
S-F	14	15.1%	12	13.2%	13	13.7%	14	15.2%
S-L	28	30.1%	27	29.7%	25	26.3%	29	31.5%
T-I	12	12.9%	13	14.3%	11	11.6%	9	9.8%
T-M	4	4.3%	4	4.4%	5	5.3%	4	4.3%
