# Supplementary material for: Complete Plastid Genomes of Nine Species of Ranunculeae (Ranunculaceae) and Their Phylogenetic Inferences
Source: Genes (Basel). 2023 Nov 27;14(12):2140. doi: 10.3390/genes14122140 (PMC10742492; doi:10.3390/genes14122140)
Supplement: Supplementary file 1 [file genes-14-02140-s001.zip › Table S3.pdf]

Table S3a. Base composition in the *Ranunculus bungei* plastid genome.

	T %	C %	A%	G%	Length (bp)
**Total**	31.4	19.3	30.7	18.5	156082
**LSC**	32.5	18.5	31.5	17.5	85430
**SSC**	34.7	16.8	34.0	14.4	19948
**IR**	28.1	20.9	28.5	22.5	25352
**tRNA**	23.0	26.6	24.0	26.4	2789
**rRNA**	22.3	27.7	22.3	27.7	9050
**Protein Coding genes**	31.5	19.7	30.3	18.5	79103

Table S3b. Base composition in the *R. pekinense* plastid genome.

	T %	C %	A%	G%	Length (bp)
**Total**	31.4	19.3	30.7	18.5	156139
**LSC**	32.5	18.5	31.5	17.5	85431
**SSC**	34.8	16.9	33.9	14.4	19956
**IR**	28.1	20.9	28.5	22.5	25376
**tRNA**	23.1	26.5	24.0	26.4	2715
**rRNA**	22.3	27.7	22.3	27.7	9050
**Protein Coding genes**	31.4	19.7	30.3	118.5	79103

Table S3c. Base composition in the *R. trichophyllus* DR plastid genome.

	T %	C %	A%	G%	Length (bp)
**Total**	31.4	19.3	30.8	18.5	158314
**LSC**	32.5	18.6	31.3	17.5	84945
**SSC**	32.7	17.3	34.3	15.7	22784
**IR**	29.3	22.1	28.1	20.5	27867
**tRNA**	23.1	26.5	24.0	26.4	2715
**rRNA**	22.3	27.7	22.3	27.7	9050

**Protein Coding genes**	31.4	19.8	30.3	18.5	79100

Table S3d. Base composition in the *R. monophyllus* plastid genome.

	T %	C %	A%	G%	Length (bp)
**Total**	31.3	19.4	30.8	18.6	155973
**LSC**	32.4	18.6	31.4	17.6	85345
**SSC**	34.4	16.8	34.2	14.6	19790
**IR**	28.5	22.4	28.1	21.0	25419
**tRNA**	23.0	26.6	24.1	26.3	2715
**rRNA**	22.3	27.7	22.3	27.7	9050
**Protein Coding genes**	31.4	19.7	30.3	18.6	79144

Table S3e. Base composition in the *R. polyrhizos* plastid genome.

	T %	C %	A%	G%	Length (bp)
**Total**	31.4	19.3	30.8	18.5	156000
**LSC**	32.5	18.5	31.4	17.6	85414
**SSC**	34.4	16.8	34.2	14.6	19677
**IR**	28.5	22.4	28.1	21.0	25455
**tRNA**	23.0	26.6	24.1	26.3	2715
**rRNA**	22.3	27.7	22.3	27.7	9050
**Protein Coding genes**	31.4	19.7	30.3	18.6	79120

Table S3f. Base composition in the *R. tanguticus* plastid genome.

	T %	C %	A%	G%	Length (bp)
**Total**	31.4	19.4	30.7	18.5	156186
**LSC**	32.5	18.5	31.4	17.6	85627
**SSC**	34.4	16.9	34.1	14.6	19785

**IR**	28.4	22.5	28.1	21.0	25387
**tRNA**	23.0	26.6	24.1	26.3	2715
**rRNA**	22.3	27.7	22.3	27.7	9050
**Protein Coding genes**	31.4	19.7	30.3	18.6	79127

Table S3g. Base composition in the *R. mongolicus* plastid genome.

	**T %**	**C %**	**A%**	**G%**	**Length (bp)**
**Total**	31.4	19.3	30.7	18.5	158309
**LSC**	32.5	18.6	31.4	17.5	84974
**SSC**	34.3	16.8	34.5	14.5	17637
**IR**	28.2	20.5	29.3	22.1	27849
**tRNA**	23.1	26.5	24.0	26.4	2715
**rRNA**	22.3	27.7	22.3	27.7	9050
**Protein Coding genes**	31.4	19.8	30.3	18.5	79082

Table S3h. Base composition in the *R. trichophyllus* ZB plastid genome.

	**T %**	**C %**	**A%**	**G%**	**Length (bp)**
**Total**	31.4	19.3	30.8	18.5	158304
**LSC**	32.5	18.6	31.3	17.5	84949
**SSC**	34.3	16.8	34.5	14.4	17619
**IR**	28.1	20.5	29.3	22.1	27868
**tRNA**	23.1	26.5	24.0	26.4	2715
**rRNA**	22.3	27.7	22.3	27.7	9050
**Protein Coding genes**	31.4	19.8	30.3	18.5	79100

Table S3i. Base composition in the *Ceratocephala testiculata* S3 plastid genome.

	**T %**	**C %**	**A%**	**G%**	**Length (bp)**

**Total**	31.1	19.6	30.5	18.8	150820
**LSC**	32.2	18.9	31.1	17.9	83575
**SSC**	33.9	17.2	33.8	15.1	18909
**IR**	28.8	22.8	27.5	20.9	24168
**tRNA**	23.1	26.4	24.1	26.4	2714
**rRNA**	22.4	27.6	22.4	27.6	9052
**Protein Coding genes**	31.2	20.0	30.1	18.8	75944

Table S3j. Base composition in the *C. testiculata* S46 plastid genome.

	**T %**	**C %**	**A%**	**G%**	**Length (bp)**
**Total**	31.1	19.6	30.5	18.8	150820
**LSC**	32.2	18.9	31.1	17.9	83575
**SSC**	33.9	17.2	33.8	15.1	18909
**IR**	28.8	22.8	27.5	20.9	24168
**tRNA**	23.1	26.4	24.1	26.4	2714
**rRNA**	22.4	27.6	22.4	27.6	9052
**Protein Coding genes**	31.2	20.0	30.1	18.8	75938

Table S3k. Base composition in the *Halerpestes tricuspis* plastid genome.

	**T %**	**C %**	**A%**	**G%**	**Length (bp)**
**Total**	31.3	19.3	30.9	18.6	158344
**LSC**	32.4	18.5	31.5	17.6	86441
**SSC**	33.6	16.9	34.1	15.4	21735
**IR**	28.3	22.5	28.3	20.9	25084
**tRNA**	23.1	26.5	24.1	26.4	2715
**rRNA**	22.3	27.7	22.3	27.7	9050
**Protein Coding genes**	31.4	19.7	30.3	18.6	78734
