# Supplementary material for: Complete Plastid Genomes of Nine Species of Ranunculeae (Ranunculaceae) and Their Phylogenetic Inferences
Source: Genes (Basel). 2023 Nov 27;14(12):2140. doi: 10.3390/genes14122140 (PMC10742492; doi:10.3390/genes14122140)
Supplement: Supplementary file 1 [file genes-14-02140-s001.zip › Table S4.pdf]

Table S4. Simple sequence repeats (SSRs) for the 11 newly sequenced Ranunculeae samples.

Genomes	Repeat units	Number	Total	Percentage (%)	Location			Region		
					Intron	IGS	CDS	LSC	SSC	IR
Ceratocephala testiculata S3	A/T	25	47	53.2		15	10	15	8	2
	AT/TA	8		17.0	1	7		7	1	
	AAT/TTA	2		4.3		2		2		
	ACC/TGG	1		2.1			1	1		
	AAAG/TTTC	1		2.1	1			1		
	AAAT/AATA	1		2.1		1		1		
	AATC/TTGA	1		2.1			1		1	
	AATG/TGAA	1		2.1		1		1		
	AATT/TTAA	3		6.4	1	2		2	1	
	AAAAG/TTTTC	1		2.1			1		1	
	AATAC/ATTGT	1		2.1		1		1		
	ATATC/CATAT	2		4.3		2		1	1	
Ceratocephala testiculata S46	A/T	26	48	54.2		17	9	16	8	2
	AT/TA	8		16.7	1	7		7	1	
	AAT/TTA	2		4.2		2		2		
	ACC/TGG	1		2.1			1	1		
	AAAG/TTTC	1		2.1		1		1		
	AAAT/AATA	1		2.1		1		1		
	AATC/TTGA	1		2.1			1		1	
	AATG/TGAA	1		2.1		1		1		
	AATT/TTAA	3		6.3	1	2		2	1	
	AAAAG/TTTTC	1		2.1			1		1	

	AATAC/ATTGT	1		2.1		1		1		
	ATATC/CATAT	2		4.2		2		1	1	
Halerpestes tricuspis	A/T	49		70.0	10	30	9	38	9	2
	AT/TA	9		12.9	1	8		7	2	
	AAT/TTA	1		1.4		1		1		
	ACC/TGG	1		1.4			1	1		
	AAAC/ACAA	1		1.4			1		1	
	AAAG/TTTC	2		2.9	1	1		2		
	AAAT/AATA	1	70	1.4		1		1		
	AATG/TGAA	1		1.4			1		1	
	AATT/TTAA	1		1.4		1		1		
	ATAG/ATCT	1		1.4	1			1		
	AAACC/TTGGT	1		1.4		1		1		
	AATAT/ATTAT	1		1.4		1		1		
	AAATCG/ATTTCG	1		1.4		1		1		
Ranunculus bungei	A/T	31		55.4	4	16	11	22	7	2
	AT/TA	9		16.1	1	6	2	7		2
	AAT/TTA	2		3.6		2		2		
	AAC/TTG	1		1.8			1			1
	AAG/TTC	3		5.4		2	1		1	2
	AAAC/ACAA	1	56	1.8			1		1	
	AAAG/TTTC	1		1.8	1			1		
	AAAT/AATA	1		1.8		1		1		
	AAGT/CATT	2		3.6		1	1	1	1	
	AATT/TTAA	2		3.6		2		2		
	AAAAT/AAATA	1		1.8		1		1		

	ACTCTG/AGTCTC	2		3.6		2				2
Ranunculus mongolicus	A/T	36		58.1	4	23	9	28	4	4
	G/C	1		1.6	1			1		
	AT/TA	7		11.3	1	6		7		
	AG/TC	2		3.2			2			2
	AAT/TTA	4		6.5		4		2		2
	AAG/TTC	2		3.2		2				2
	AAAC/ACAA	1	62	1.6			1		1	
	AAAG/TTTC	1		1.6	1			1		
	AAAT/AATA	4		6.5		4		2		2
	AAGT/CATT	1		1.6		1		1		
	AATG/TGAA	1		1.6			1		1	
	AATT/TTAA	2		3.2		2		2		
Ranunculus monophyllus	A/T	32		58.2	8	14	10	23	8	1
	G/C	1		1.8		1		1		
	AT/TA	6		10.9	1	5		5	1	
	AAC/TTG	1		1.8			1			1
	AAT/TTA	4		7.3	1	3		3	1	
	AAAG/TTTC	1		1.8	1			1		
	AAAT/AATA	2	55	3.6	1	1		2		
	AATC/TTGA	1		1.8		1		1		
	AATG/TGAA	2		3.6		1	1	1	1	
	AATT/TTAA	1		1.8		1		1		
	AAATT/ATTAA	1		1.8		1		1		
	AATAT/TATAT	1		1.8		1		1		
	AATGC/GCAAT	2		3.6			2			2

Ranunculus pekinense	A/T	33	61	54.1	2	20	11	24	7	2
	AT/TA	7		11.5	1	6		7		
	AG/TC	2		3.3			2			2
	AAC/TTG	1		1.6			1			1
	AAG/TTC	6		9.8		5	1		1	5
	AAT/TTA	2		3.3		2		2		
	AAAC/ACAA	1		1.6			1		1	
	AAAG/TTTC	1		1.6	1			1		
	AAAT/AATA	1		1.6		1		1		
	AAGT/CATT	1		1.6		1		1		
	AATG/TGAA	1		1.6			1		1	
	AATT/TTAA	2		3.3		2		2		
	AAAAT/AAATA	1		1.6		1		1		
	ACTCTG/AGTCTC	2		3.3		2				2
Ranunculus polyrhizos	A/T	31	51	60.8	6	17	8	25	6	
	AT/TA	6		11.8	1	5		4	2	
	AAC/TTG	1		2.0			1			1
	AAT/TTA	4		7.8	1	3		3	1	
	AAAG/TTTC	1		2.0	1			1		
	AAAT/AATA	1		2.0	1			1		
	AATG/TGAA	2		3.9		1	1	1	1	
	AATT/TTAA	2		3.9		2		2		
	AAATT/ATTAA	1		2.0		1		1		
	AATGC/GCAAT	2		3.9			2			2
Ranunculus tanguticus	A/T	30	49	61.2	6	14	10	22	7	1
	AT/TA	6		12.2	1	5		5	1	

	AAC/TTG	1		2.0			1			1
	AAT/TTA	5		10.2	1	4		4	1	
	AAAG/TTTC	1		2.0	1			1		
	AATC/TTGA	1		2.0		1		1		
	AATG/TGAA	2		4.1		1	1	1	1	
	AATT/TTAA	2		4.1		2		2		
	AAATG/TTACT	1		2.0		1		1		
Ranunculus trichophyllus DR	A/T	42		61.8	6	26	10	32	4	6
	G/C	1		1.5	1			1		
	AT/TA	7		10.3	1	6		7		
	AG/TC	2		2.9			2			2
	AAG/TTC	2		2.9		2				2
	AAT/TTA	4		5.9		4		2		2
	AAAC/ACAA	1	68	1.5			1		1	
	AAAG/TTTC	1		1.5	1			1		
	AAAT/AATA	3		4.4		3		1		2
	AAGT/CATT	1		1.5		1		1		
	AATG/TGAA	1		1.5			1		1	
	AATT/TTAA	2		2.9		2		2		
	AAAAT/AAATA	1		1.5	1			1		
Ranunculus trichophyllus ZB	A/T	42		61.8	6	26	10	32	4	6
	G/C	1		1.5	1			1		
	AT/TA	7	68	10.3	1	6		7		
	AG/TC	2		2.9			2			2
	AAG/TTC	2		2.9		2				2
	AAT/TTA	4		5.9		4		2		2

AAAC/ACAA	1	1.5			1		1	
AAAG/TTTC	1	1.5	1			1		
AAAT/AATA	3	4.4		3		1		2
AAGT/CATT	1	1.5		1		1		
AATG/TGAA	1	1.5			1		1	
AATT/TTAA	2	2.9		2		2		
AAAAT/AAATA	1	1.5	1			1		
