# Supplementary material for: Complete Plastid Genomes of Nine Species of Ranunculeae (Ranunculaceae) and Their Phylogenetic Inferences
Source: Genes (Basel). 2023 Nov 27;14(12):2140. doi: 10.3390/genes14122140 (PMC10742492; doi:10.3390/genes14122140)
Supplement: Supplementary file 1 [file genes-14-02140-s001.zip › Table S5.pdf]

Table S5. The potential positive selection test based on the branch-site model of Ranunculeae.

Gene	Alternative hypothesis			Null hypothesis			2Δl	df	p-value	Positive selection site(BEB)
name	InL	np	ω	InL	np	ω				
accD	-2575.4624	65	1.51268	-2576.0630	64	1.60322	1.201166	1	0.273089	7 S 0.977*; 17 Y 0.978*; 26 L 0.968*; 28 S 0.956*; 32 L 0.971; 36 Q 0.998**; 38 Q 0.987*; 41 L 0.951*; 42 C 0.999**; 44 W 0.994**; 49 N 0.964*; 50 W 0.956*; 57 S 0.997**; 163 N 0.967*; 164 A 0.998**; 172 P 0.977*; 180 R 0.982*; 254 V 0.976*; 268 A 0.984*; 272 R 0.955*
atpA	-3465.5064	65	0.07107	-3465.5145	64	0.07164	0.016292	1	0.898434	
atpB	-3295.9048	65	0.11638	-3299.0096	64	0.09198	6.209544	1	0.012706	
atpE	-886.09371	65	0.07947	-886.76432	64	0.06362	1.341232	1	0.246817	
atpF	-1307.8898	65	0.24521	-1307.9904	64	0.26109	0.201234	1	0.653727	89 A 0.962*; 107 L 0.976*
atpH	-450.25162	65	0.0001	-450.25162	64	0.0001	0	1	1	
atpI	-1556.232	65	0.07784	-1556.7623	64	0.06319	1.06069	1	0.303058	
ccsA	-3465.1459	65	0.25825	-3465.3561	64	0.25485	0.420462	1	0.516707	168 N 0.991**
cemA	-1916.6582	65	0.26388	-1916.8997	64	0.27684	0.482978	1	0.487077	
matK	-5307.8173	65	0.39308	-5309.499	64	0.35405	3.363272	1	0.066665	
ndhB	-2402.7960	65	0.23563	-2403.0591	64	0.22203	0.526224	1	0.468199	
ndhC	-840.5609	65	0.06866	-842.8564	64	0.1186	4.590842	1	0.032143	
ndhE	-791.7393	65	0.1139	-792.1273	64	0.12714	0.776134	1	0.378326	
ndhF	-8014.3722	65	0.1927	-8015.4328	64	0.20198	2.121182	1	0.145274	602 Q 0.957*; 625 R 0.979*
ndhG	-1515.9022	65	0.2849	-1521.7343	64	0.19713	11.66421	1	0.000637	
ndhH	-3010.9664	65	0.0903	-3012.3148	64	0.08225	2.696832	1	0.100548	
ndhI	-1340.6988	65	0.1280	-1340.7530	63	0.13326	0.108346	2	0.947268	
ndhJ	-1054.0633	65	0.0649	-1057.1720	64	0.04614	6.21738	1	0.01265	
petA	-2468.1783	65	0.1065	-2468.9043	64	0.09818	1.45199	1	0.228209	
petB	-1305.3526	65	0.0113	-1306.1533	64	0.01856	1.601492	1	0.205692	
petD	-1141.6069	65	0.0976	-1143.6760	63	0.06402	4.138194		0.1263	164 P 0.985*
petG	-232.6066	65	0.0483	-233.1290	64	0.02795	1.044854	1	0.306695	
petL	-195.05554	65	0.0610	-195.66676	64	0.04362	1.22243	1	0.268884	
petN	-117.03045	65	0.0001	-117.03045	64	0.0001	0	1	1	
psaA	-4729.9671	65	0.01463	-4731.1721	64	0.0194	2.41015	1	0.120551	
psaB	-4372.8062	65	0.01737	-4373.7591	64	0.02066	1.905768	1	0.167434	
psaC	-564.13122	65	0.0001	-566.28432	64	0.00484	4.306198	1	0.037974	
psaI	-241.87836	65	0.24206	-241.88125	64	0.24707	0.005772	1	0.93944	
psaJ	-283.07602	65	0.09359	-283.81674	64	0.13563	1.481436	1	0.22355	
psbA	-2082.286	65	0.02251	-2082.3302	64	0.02081	0.088396	1	0.766226	
psbC	-2795.9512	65	0.02125	-2796.0059	64	0.02234	0.109486	1	0.74073	

psbD	-1989.939	65	0.01955	-1989.4553	64	0.02341	-0.96743	1	0.325322	
psbE	-463.81161	65	0.03208	-464.67621	64	0.01308	1.7292	1	0.188513	
psbF	-192.99787	65	0.0001	-192.99787	64	0.0001	0	1	1	
psbH	-624.28765	65	0.26604	-625.51052	64	0.17428	2.445724	1	0.117846	
psbI	-199.52055	65	0.0001	-199.52055	64	0.0001	0	1	1	
psbJ	-227.42673	65	0.33001	-228.1227	64	0.19896	1.391946	1	0.238077	
psbK	-411.91192	65	0.17991	-412.23882	64	0.15691	0.653812	1	0.418753	
psbL	-174.86292	65	0.0001	-174.87262	64	0.0001	0.01941	1	0.889198	
psbM	-163.06198	65	0.06513	-163.37262	64	0.04641	0.621282	1	0.430571	
psbN	-233.24717	65	0.0001	-233.24717	64	0.0001	0	1	1	
psbT	-204.12814	65	0.0001	-204.41989	64	0.02262	0.583494	1	0.444946	
rbcL	-3221.9468	65	0.12162	-3222.9628	64	0.136	2.03203	1	0.154015	30 T 0.981*; 251 I 0.992**; 328 S 0.992**
rpl14	-718.84085	65	0.06987	-720.53206	64	0.05497	3.382426	1	0.065895	
rpl16	-758.57004	65	0.12553	-758.5841	64	0.12733	0.028128	1	0.866808	
rpl20	-937.54875	65	0.3437	-937.85106	64	0.37266	0.604626	1	0.436818	
rpl23	-397.58738	65	0.18532	-397.58698	64	0.18627	-0.00079	1	0.977549	
rpl33	-474.47258	65	0.1113	-475.57912	64	0.15391	2.213068	1	0.136846	
rpl36	-274.89508	65	0.0001	-275.89803	64	0.026	2.005898	1	0.156689	
rpoA	-2619.694	65	0.2009	-2619.6943	64	0.20123	0.000578	1	0.980819	237 Q 0.967*
rpoB	-7576.4396	65	0.1197	-7576.601	64	0.12271	0.322822	1	0.569917	
rpoC1	-4709.2816	65	0.17983	-4710.3408	64	0.16025	2.118484	1	0.145531	
rpoC2	-11009.538	65	0.20578	-11010.871	64	0.2152	2.665184	1	0.102566	491 S 0.997**; 934 L 0.968*
rps2	-1603.8961	65	0.23607	-1606.8214	64	0.17538	5.850678	1	0.015571	
rps3	-1780.3431	65	0.16321	-1781.3902	64	0.17269	2.094284	1	0.147851	
rps7	-809.02224	65	0.83445	-810.46347	64	0.57434	2.882454	1	0.089549	
rps8	-850.00316	65	0.12626	-851.42189	64	0.13949	2.837472	1	0.092089	
rps11	-1092.6909	65	0.23273	-1093.4355	64	0.19382	1.489216	1	0.222338	
rps12	-165.52404	65	0.07478	-165.524	64	0.07477	-7.6E-05	1	0.994954	
rps14	-647.47535	65	0.33459	-647.5806	64	0.30849	0.210502	1	0.646374	
rps15	-830.73094	65	0.29433	-833.00005	64	0.23913	4.538228	1	0.033146	
rps16	-655.38107	65	0.34369	-657.27723	64	0.26342	3.79233	1	0.051488	
rps18	-196.26966	65	0.20807	-196.81775	64	0.17823	1.096186	1	0.295105	
rps19	-148.09896	65	0.16511	-148.93131	64	0.11658	1.664694	1	0.196971	
ycf2	-8400.9354	65	0.67708	-8404.084	64	0.5723	6.297032	1	0.012094	430 F 0.957*
ycf3	-981.28672	65	0.03939	-982.30863	64	0.03015	2.043814	1	0.152826	
ycf4	-1500.9204	65	0.22258	-1502.196	64	0.18777	2.551128	1	0.110216	

* : P>0.900, **: P>0.950.
